# Utility of Oral Swab Sampling for Ebola Virus Detection in Guinea Pig Model

**DOI:** 10.3201/eid2110.150840

**Published:** 2015-10

**Authors:** Jessica R. Spengler, Ayan K. Chakrabarti, JoAnn D. Coleman-McCray, Brock E. Martin, Stuart T. Nichol, Christina F. Spiropoulou, Brian H. Bird

**Affiliations:** Centers for Disease Control and Prevention, Atlanta, Georgia, USA

**Keywords:** Ebola, viral hemorrhagic fever, qRT-PCR, oral swab, guinea pig, wild-type Ebola virus, viruses

## Abstract

To determine the utility of oral swabs for diagnosing infection with Ebola virus, we used a guinea pig model and obtained daily antemortem and postmortem swab samples. According to quantitative reverse transcription PCR analysis, the diagnostic value was poor for antemortem swab samples but excellent for postmortem samples.

Ebola virus (EBOV) causes Ebola virus disease (EVD), which results in a high number of deaths in humans. EBOV is the etiologic agent of the ongoing EVD outbreak in West Africa. Nonadapted EBOV causes disease in nonhuman primates, but adaptation is required for the virus to cause disease in rodent models (*1*–*4*). Fatal disease has been observed in 20% of guinea pigs infected with wild-type (WT) nonadapted EBOV, but a uniformly lethal guinea pig–adapted EBOV isolate was found to have developed after a limited number of serial infection passages in guinea pigs (*3**,**5**,**6*).

Real-time quantitative reverse transcription PCR (qRT-PCR) is used to detect EBOV in the current West Africa outbreak. Appropriate sample collection and knowledge of interpreting results on the basis of specimen type are essential for accurate triage of patients thought to have EVD. Oral swab sampling for postmortem EBOV diagnosis has been supported by use of a nonhuman primate model (*7*), and oral swab sampling for antemortem EVD diagnosis has been a major consideration in the current outbreak because collection of swab samples is less invasive than collection of serum samples and poses a much lower risk of transmitting EBOV to the person obtaining the sample than traditional phlebotomy. However, the utility of oral swabs for antemortem testing has not been investigated in detail under controlled experimental conditions. In addition, Bausch et al. have suggested that the oral milieu, such as saliva composition and oral cavity tissue structure, may potentially inhibit diagnostic capabilities of oral swab sampling (*8*).

Wong et al. have shown that oral swabbing can be used to detect virus and shedding in guinea pigs at isolated intervals after infection (*9*). We investigated oral swab sampling as an antemortem means of diagnosing EVD and used qRT-PCR to detect EBOV RNA in daily oral swab samples obtained from guinea pigs infected with guinea pig–adapted EBOV (GP-EBOV) and with WT-EBOV.

## The Study

Procedures and experiments described herein were approved by the Centers for Disease Control and Prevention (CDC) Institutional Animal Care and Use Committee and conducted in strict accordance with the Guide for the Care and Use of Laboratory Animals (*10*). CDC is a fully accredited research facility of the Association for Assessment and Accreditation of Laboratory Animal Care International.

Healthy adult male and female strain 13/N guinea pigs, 1.0–2.5 years of age, were housed in a Biosafety Level 4 laboratory in microisolator cage systems filtered with high-efficiency particulate arrestance filters. Groups of 5 animals, distributed proportionally by age and sex, were inoculated intraperitoneally with a 50% tissue culture infectious dose (TCID_50_) at low (5 TCID_50_) or high (5,000 TCID_50_) levels of GP-EBOV-Mayinga, or with 5 × 10^5^ TCID_50_ of either the WT-EBOV-Mayinga 1976 variant (Ebola virus/*H. sapiens*-tc/COD/1976/Yambuku-Mayinga) or the WT-EBOV-Makona 2014 variant (Ebola virus/*H. sapiens*-tc/LBR/2014/Makona; GenBank accession no. KP178538). To serve as negative controls, 3 animals were inoculated intraperitoneally with Dulbecco’s Modified Eagle’s Medium. Animals were monitored for signs of clinical illness, and body weight and temperature readings were obtained daily. Oral swab samples were collected daily for isolation of RNA and analyzed by qRT-PCR. Postmortem oral swab samples were obtained from 10 animals that were euthanized because of severe clinical illness consistent with EBOV. Carcasses of the dead animals were kept in an incubator at 30°C to simulate conditions in equatorial Africa. Samples were obtained from 9 of the 10 animals up to 5 days after death and from 1 animal at 2 days after death. In addition to oral swab samples, paired blood samples were collected from the cranial vena cava of anesthetized animals at 3 days postinfection (dpi) and by cardiac puncture at the time of death for euthanized animals.

Low and high doses of GP-EBOV-Mayinga were uniformly lethal. Clinical illness was delayed in 1 animal in the high-dose group; the animal was euthanized at 12 dpi, but all other animals were euthanized by 9 dpi. One animal infected with nonadapted WT-EBOV-Mayinga was euthanized at 9 dpi because of clinical illness. No severe clinical illness developed in any of the other animals infected with WT-EBOV-Mayinga or WT-EBOV-Makona (Figure, panel A). Fever developed in all animals infected with low- and high-dose GP-EBOV-Mayinga, in 20% of animals infected with WT-EBOV-Makona or WT-EBOV-Mayinga, and in none of the negative control animals. Hypothermia, typical during the terminal phases of many disease processes, was observed in animals with end-stage EVD (Figure, panel B). Substantial weight loss (>15%) was observed in all febrile animals (Figure, panel C). The 1 animal infected with WT-EBOV-Makona that showed clinical sign experienced transient fever and weight loss but started to regain weight by 9 dpi.

**Figure:**
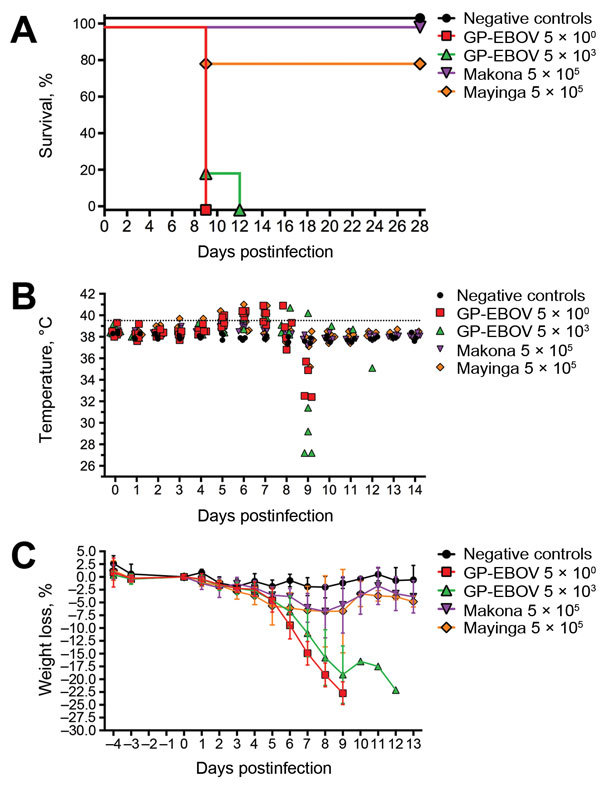
Clinical course of guinea pigs infected with guinea pig–adapted Ebola virus (GP-EBOV), wild-type EBOV Makona, and wild-type EBOV Mayinga, by number of days postinfection. A) Percentage of animals that survived. B) Subcutaneous microchip temperature. Dotted line indicates upper limit of reference temperature range for guinea pigs. C) Weight loss from 0 days postinfection.

Oral swab samples were analyzed by qRT-PCR targeting the EBOV nucleoprotein gene; 18s ribosomal RNA levels were also analyzed to serve as a sampling control. EBOV RNA abundance was calculated by comparing the cycle threshold values to an in vitro–transcribed small-segment RNA standard of known copy number. All oral swab samples that were collected 0–4 dpi were negative for EBOV nucleoprotein RNA (Table). At 3 dpi, blood samples from 7 (41%) of 17 infected animals from which blood samples could be obtained were positive for EBOV, but no viral RNA was detected in any of the paired oral swab samples. The earliest detection of EBOV RNA by oral swabbing was at 5 dpi in an animal infected with WT-EBOV-Mayinga. At 6 dpi, coinciding with the time of overt clinical signs of disease (i.e., fever, weakness, anorexia, and ruffled fur), qRT-PCR of oral swab samples detected EBOV RNA in 8 (73%) of 11 animals in which fatal illness developed and in 10 (50%) of 20 infected animals. EBOV RNA was detected by qRT-PCR in all postmortem swab samples.

**Table:** qRT-PCR results for EBOV nucleoprotein from guinea pig oral swab and blood samples collected, by number of days postinfection*

ID	Virus type	Dose, TCID_50_/mL	Sample type, blood or oral/blood†													
			D0	D1	D2	D3	D4	D5	D6	D7	D8	D9	D10	D11	D12	D14
1	GP-EBOV	5.0 × 10^0^	–	–	–	–/–	–	–	+	+	++	+++/++++	NS	NS	NS	NS
2	GP-EBOV	5.0 × 10^0^	–	–	–	–/–	–	–	+	++	+++	+++/+++++	**++++**	**++++**	**+++**	**++++**
3	GP-EBOV	5.0 × 10^0^	–	–	–	–/–	–	–	+	+++	++++	++++/+++++	**+++++**	**++++**	**+++**	**++++**
4	GP-EBOV	5.0 × 10^0^	–	–	–	–/–	–	–	++	+++	+++	++++/+++++	**++++**	**++++**	**+++**	**++++**
5	GP-EBOV	5.0 × 10^0^	–	–	–	–/NS	–	–	–	++	+++	++++/+++++	**++++**	**++++**	**++++**	**++++**
6	GP-EBOV	5.0 × 10^3^	–	–	–	–/–	–	–	+	+++	+++	+++/++++	**+++**	**+++**	**++**	**+++**
7	GP-EBOV	5.0 × 10^3^	–	–	–	–/+	–	–	++	+++	+++	+++/+++++	**++++**	**++++**	**+++**	**++++**
8	GP-EBOV	5.0 × 10^3^	–	–	–	–/NS	–	–	++	++	+++	+++/++++	**++++**	**+++++**	**+++**	**++++**
9	GP-EBOV	5.0 × 10^3^	–	–	–	–/–	–	–	–	–	–	–	+	+	+/+++	**++**
10	GP-EBOV	5.0 × 10^3^	–	–	–	–/+++	–	–	++	++	+++	++++/+++	**++++**	**+++++**	**+++**	**+++**
11	WT-Makona	5.0 × 10^5^	–	–	–	–/–	–	–	–	–	–	–	–	–	–	–
12	WT-Makona	5.0 × 10^5^	–	–	–	–/++	–	–	–	–	–	–	–	–	–	–
13	WT-Makona	5.0 × 10^5^	–	–	–	–/–	–	–	–	–	–	–	–	–	–	–
14	WT-Makona	5.0 × 10^5^	–	–	–	–/++	–	–	+	+	+	–	–	–	–	–
15	WT-Makona	5.0 × 10^5^	–	–	–	–/+	–	–	–	–	–	–	–	–	–	–
16	WT-Mayinga	5.0 × 10^5^	–	–	–	–/++	–	–	–	–	–	–	–	–	–	–
17	WT-Mayinga	5.0 × 10^5^	–	–	–	–/–	–	–	+	–	–	–	–	–	–	–
18	WT-Mayinga	5.0 × 10^5^	–	–	–	–/NS	–	+	–	+	+	–	–	–	–	–
19	WT-Mayinga	5.0 × 10^5^	–	–	–	–/–	–	–	–	–	–	–	–	–	–	–
20	WT-Mayinga	5.0 × 10^5^	–	–	–	–/+++	–	–	–	++	+	+/+	**++**	**++**	**++**	**+++**
21	Neg control	DMEM	–	–	–	–/NS	–	–	–	–	–	–	–	–	–	–
22	Neg control	DMEM	–	–	–	–/–	–	–	–	–	–	–	–	–	–	–
23	Neg control	DMEM	–	–	–	–/–	–	–	–	–	–	–	–	–	–	–

*Boldface indicates postmortem samples. Gray shading indicates period of overt clinical disease (days 6–9 postinfection). D, days postinfection; DMEM, Dulbecco's Modified Eagle's medium; EBOV, Ebola virus; GP, guinea pig–adapted; ID, identification number; Neg, negative; NS, not sampled; qRT-PCR, quantitative reverse transcription PCR; TCID_50_, 50% tissue culture infectious dose; WT, wild-type. †EBOV RNA copies/mL: –, negative; +, 10^0^–10^1^; ++, 10^2^; +++; 10^3^; ++++, 10^4^; +++++, 10^5^–10^6^.

## Conclusions

Our data suggest that oral swab samples obtained early in the course of infection, before death, are not a reliable method for diagnosing infection with EBOV. Paired oral swab and blood samples collected at 3 dpi and at time of euthanasia showed that sensitivity of oral swab samples was low compared with the sensitivity of traditional blood samples. Testing of oral swab samples did not indicate infection until 3 days after EBOV RNA was detectable in blood samples, with the exception of 1 animal in which oral swab samples revealed viral RNA 2 days after the blood sample. At the time of overt clinical disease, the utility of oral swab samples for diagnostics improved but was not completely consistent with infection until postmortem analysis. Our studies also enabled us to investigate whether the virulence of the WT-EBOV-Makona variant in guinea pigs was as low as that of the prototypic WT-EBOV-Mayinga variant. As shown in previous studies (*3**,**5**,**6*), WT-EBOV is less pathogenic than GP-EBOV, regardless of variant, in this animal model.

Investigating the utility of oral swab samples for diagnosing EVD in humans is challenging because paired blood and oral swab samples are rarely available and because the timing of sample collection relative to onset of disease and course of infection is often estimated. Although EVD in the nonhuman primate model mimics many aspects of the disease in humans, sampling from nonhuman primates in an experimental setting is problematic because of the species’ temperament, which requires anesthesia during specimen collection and venipuncture. The guinea pig model of EVD (*3**,**5**,**6*) offers the convenience of daily oral swab sampling without the need for anesthesia.

Although suggestive, as with any animal model system, when extrapolating these data to human diagnostics, the effect of potential differences in oral milieus (e.g., saliva composition and oral cavity tissue structure) must be considered. In the future, additional studies that use paired oral swab and blood samples from humans would provide information for continued discussion of antemortem swab sampling as a useful diagnostic modality of EVD in humans.

Our data support the use of oral swab samples as a sensitive modality for postmortem diagnostics; however, the utility of oral swab samples under field conditions, especially those collected before death, may decrease because of inherent problems with sampling techniques and specimen handling conditions (i.e., delays in transport and storage at typically high ambient temperatures). Despite these considerations, oral swab sample collection could be a useful sampling strategy for humans and animals with unknown causes of death when EVD is suspected and when other types of samples are more prohibitive to obtain.
